# Exploring the Impact of Authentic Leadership on Nurses' Organisational Citizenship Behaviour: Organisational Silence as a Mediator

**DOI:** 10.1111/jan.70005

**Published:** 2025-07-01

**Authors:** Hyun Ju Lho, Ja Kyung Seo, Seung Eun Lee

**Affiliations:** ^1^ Severance Hospital, College of Nursing, Graduate School Yonsei University Seoul South Korea; ^2^ Psychological Science Innovation Institute, Department of Psychology Yonsei University Seoul South Korea; ^3^ Department of Psychology and Business Korea Military Academy Seoul South Korea; ^4^ College of Nursing, Mo‐Im KIM Nursing Research Institute Yonsei University Seoul South Korea

**Keywords:** acquiescent silence, authentic leadership, defensive silence, nurses, organisational citizenship behaviour, organisational silence, prosocial silence

## Abstract

**Aim:**

To investigate the relationship between nurse managers' authentic leadership and staff nurses' organisational citizenship behaviour and to explore the mediating roles of different types of organisational silence in the relationship.

**Design:**

A cross‐sectional, correlational, descriptive design.

**Methods:**

Survey data from 216 nurses across five hospitals in South Korea were utilised. We used structural equation modelling to examine the relationships between authentic leadership, organisational silence and organisational citizenship behaviour.

**Results:**

Authentic leadership was positively associated with organisational citizenship behaviour and prosocial silence and was negatively associated with acquiescent and defensive silence. Acquiescent silence negatively impacted organisational citizenship behaviour, whereas prosocial silence had a positive effect. Defensive silence showed no significant relationship with organisational citizenship behaviour. Authentic leadership's indirect effects on organisational citizenship behaviour were mediated by acquiescent and prosocial silence.

**Conclusion:**

Nurse managers' authentic leadership enhances organisational citizenship behaviour by reducing acquiescent and defensive silence and fostering prosocial silence. Although prosocial silence promotes harmony and collaboration, nurse managers must also encourage open communication to maintain a positive and professional organisational culture.

**Implications for the Profession and/or Patient Care:**

Developing authentic leadership in nurse managers can improve nurses' organisational citizenship behaviour by addressing organisational silence, thereby creating a supportive and efficient healthcare environment that benefits staff as well as patients.

**Patient or Public Contribution:**

No patient or public contribution.

**Impact:**

Authentic leaders serve as positive role models who can improve employees' organisational citizenship behaviour by reducing negative forms of organisational silence. However, the mediating effect of organisational silence on the relationship between authentic leadership and organisational citizenship behaviour may vary depending on the type of silence, as different types are induced by distinct underlying motivations. Understanding these dynamics can inform strategies to improve nurses' workplace interactions and patient care outcomes.

**Reporting Method:**

Strengthening the Reporting of Observational Studies in Epidemiology guidelines.

**Summary**

- What does this paper contribute to the wider global clinical community?
  ○ Nurse managers' authentic leadership positively influences staff nurses' organisational citizenship behaviour by reducing undesirable types of organisational silence.
  ○ The mediating role of organisational silence underscores the importance of understanding different types of silence and their unique effects on workplace dynamics and nurse behaviours.

## 1 Introduction

Organisational citizenship behaviour refers to voluntary actions by employees that extend beyond their formal job responsibilities and contribute to organisational effectiveness. Such behaviours, which are often characterised by altruism, conscientiousness or civic virtue, create a supportive and dynamic work environment, which is particularly valuable in demanding sectors such as healthcare. Studies have highlighted the role of organisational citizenship behaviour in improving organisational adaptability and performance (Hermawan et al. 2020; Thompson et al. 2020), making it a critical factor in healthcare management. Despite its recognised importance, the mechanisms that foster organisational citizenship behaviour among nurses remain underexplored. For example, leadership styles, such as authentic leadership, may play a crucial role in encouraging such behaviours, while organisational silence could act as a barrier, mediating that relationship.

In South Korea, frontline nurses face considerable burdens due to nurse shortages, high patient volumes and demanding shift work conditions (Kim 2021). Moreover, Korean healthcare organisations are often shaped by hierarchical and collectivist cultural values (Lee and Lee 2024). These contextual characteristics may influence nurses' willingness to engage in organisational citizenship behaviour or voice concerns, making leadership even more critical in facilitating positive workplace behaviours. This study aimed to investigate the interplay between nurse managers' authentic leadership and nurses' organisational silence and citizenship behaviour, with the goal of achieving insights into how organisational effectiveness can be enhanced in healthcare settings.

## 2 Background

Leadership within healthcare institutions is pivotal in cultivating a workplace environment that promotes organisational citizenship behaviour (Lee and Jang 2022). Moreover, leadership styles significantly influence the degree and quality of such behaviours among nurses (Farid et al. 2020). Among various leadership styles, authentic leadership stands out as particularly impactful in healthcare settings (Lee and Jang 2022). Authentic leaders—those characterised by self‐awareness, transparency, morality and adherence to ethical standards—actively foster a culture of trust and respect within their teams (Avolio 2007). Authentic leadership's potential to influence organisational citizenship behaviour can be understood through the lens of social exchange theory (Blau 1964). According to this theory, employees who perceive their leaders as trustworthy and supportive are more likely to reciprocate with positive organisational behaviours, including organisational citizenship behaviour (Farid et al. 2020). Although this relationship has been demonstrated in sectors outside healthcare, its implications within the healthcare context remain understudied (Lee and Jang 2022).

In addition to its well‐documented influence on organisational citizenship behaviour, authentic leadership has garnered attention for its potential to mitigate organisational silence, which refers to employees' deliberate withholding of constructive feedback or concerns regarding organisational issues (Morrison 2023). Authentic leaders play a crucial role in mitigating this phenomenon by fostering an inclusive environment where employees are actively involved in setting organisational goals and are encouraged to voice their opinions on emerging challenges. Thus, authentic leaders enhance organisational performance by integrating diverse employee perspectives into the decision‐making process (Kleynhans et al. 2022). Furthermore, their unbiased processing of information and proactive solicitation of employee input can reduce the prevalence of organisational silence (Abdillah et al. 2022).

Although previous research has examined the role of authentic leadership in reducing employee silence (Abdillah et al. 2022), limited empirical evidence exists regarding its specific impact on different types of employee silence. For example, acquiescent silence is characterised by employees who withhold input because they lack hope in their ability to affect change. This type of silence may decrease under authentic leadership. When authentic leaders foster climates of trust and respect, feelings of employee resignation may diminish, while employees' confidence in their contributions may increase (Xu et al. 2023). Similarly, defensive silence—rooted in fear of repercussions or negative outcomes—may be less likely to occur under authentic leaders who create psychologically safe environments that encourage open communication. Conversely, prosocial silence is typically driven by altruistic motives, such as protecting colleagues, avoiding unnecessary conflicts, maintaining harmony or safeguarding sensitive information (Kızrak and Yeloğlu 2024; Van Dyne et al. 2003). This type of silence may become prevalent under authentic leadership, which fosters positive relationships with employees and cultivates a strong sense of organisational belonging. In such environments, empowered employees are more likely to engage in prosocial silence for the benefit of their team or organisation (Xu et al. 2023).

Persistent organisational silence not only limits opportunities to share diverse perspectives and knowledge that could benefit the organisation but also leads to indifferent employee behaviour towards the organisation. For example, employees who remain silent often show lower levels of engagement and organisational citizenship behaviour (Mao et al. 2019), which can contribute to organisational stagnation. Previous research has predominantly focused on the detrimental effects of acquiescent and defensive silence on work outcomes, including negative attitudes towards the organisation (Abd El‐Fattah Mohamed Aly et al. 2022) and reduced organisational performance (Yağar and Dökme Yağar 2023). A meta‐analysis of organisational silence indicates that acquiescent silence and defensive silence were negatively associated with organisational citizenship behaviour, while prosocial silence showed a positive relationship (Hao et al. 2022). This finding suggests that employees engaging in prosocial silence may nonetheless exhibit high organisational citizenship behaviour because their actions are driven by proactive and altruistic motives (Acaray and Akturan 2015). However, the nuanced role of prosocial silence remains underexplored.

## 3 The Study

This study examined the impact of nurse managers' authentic leadership on staff nurses' organisational citizenship behaviour. In addition, it investigated whether authentic leadership differentially influenced three types of organisational silence, each driven by distinct underlying motivations: acquiescent silence (motivated by resignation), defensive silence (motivated by fear) and prosocial silence (motivated by desire to avoid embarrassing others or to protect personal and organisational information) (Morrison 2023). To address these objectives, we formulated the following four hypotheses:

*Authentic leadership is positively associated with organisational citizenship behaviour*.

*Authentic leadership is negatively associated with acquiescent and defensive silence, whereas it is positively associated with prosocial silence*.

*Acquiescent and defensive silence is negatively associated with organisational citizenship behaviour, whereas prosocial silence is positively associated with organisational citizenship behaviour*.

*The three types of organisational silence—acquiescent, defensive, and prosocial—mediate the relationship between authentic leadership and organisational citizenship behaviour*.

## 4 Methods

### 4.1 Study Design and Sample

This cross‐sectional, descriptive correlational study utilised convenience sampling to recruit participants between May and July of 2023. First, we approached the nursing departments of five hospitals in South Korea to explain the study's purpose and obtain departmental approval for conducting the study. The inclusion criteria were as follows: participants had to be (a) full‐time nurses with at least 1 year of experience in their current work unit and (b) had worked under their current nurse manager for more than 6 months. Nurses who volunteered to participate used a survey link posted on bulletin boards and in locker rooms to access an online questionnaire. A total of 275 online questionnaires were distributed, and 245 were returned, yielding a response rate of 89.1%. After 29 incomplete questionnaires were excluded, the final sample size was 216 participants. This sample size was deemed adequate for conducting structural equation modelling (SEM), as it aligned with Kline's (2005) recommendation of a minimum sample size between 100 and 200.

### 4.2 Measures

#### 4.2.1 Outcome Variable

Organisational citizenship behaviour was measured using the Organisational Citizenship Behaviour Scale employed by Niehoff and Moorman (1993), which was later modified by Song and Seomun (2014) for use among Korean nurses. This 20‐item scale has five 4‐item subscales: altruism, courtesy, sportsmanship, conscientiousness and civic virtue. Sample items include ‘I help others who have heavy workloads’ (altruism), ‘I do not abuse the rights of others’ (courtesy), ‘I consume a lot of time complaining about trivial matters’ (sportsmanship), ‘I am always punctual’ (conscientiousness) and ‘I keep abreast of changes in the organization’ (civic virtue). Participants responded to each item on a five‐point Likert scale ranging from 1 (*strongly disagree*) to 5 (*strongly agree*), with higher scores indicating higher levels of organisational citizenship behaviour. Song and Seomun (2014) reported a Cronbach's alpha of 0.88 for the total scale. In the current study, the Cronbach's alpha was 0.83.

#### 4.2.2 Predictor Variable

Authentic leadership was measured using the 16‐item Authentic Leadership Questionnaire (Avolio 2007), which was modified and validated by Song and Seomun (2014) for use among Korean nurses. The questionnaire has four subscales: relational transparency (5 items), internalised moral perspective (4 items), balanced processing (3 items) and self‐awareness (4 items). Example items include ‘My leader encourages everyone to speak their mind’ (relational transparency), ‘My leader makes decisions based on his or her core values’ (internalised moral perspective), ‘My leader listens carefully to different points of view before coming to conclusions’ (balanced processing) and ‘My leader seeks feedback to improve interactions with others’ (self‐awareness). Participants responded to each item on a five‐point Likert scale ranging from 1 (*not at all*) to 5 (*frequently*). Higher scores indicate a higher perceived level of managerial authentic leadership. Song and Seomun (2014) reported a Cronbach's alpha of 0.96 for the total scale. In the current study, the Cronbach's alpha was 0.94.

#### 4.2.3 Mediator Variables

Organisational silence was assessed using a scale developed by Van Dyne et al. (2003), which was later validated by Kang and Go (2014) for a Korean sample. This 13‐item scale has three subscales: acquiescent silence (5 items), defensive silence (4 items) and prosocial silence (4 items). Example items include ‘I passively keep ideas about solutions to problems to myself’ (acquiescent silence), ‘I do not speak up and suggest ideas for change, based on fear’ (defensive silence) and ‘I refuse to divulge information that might harm the organization’ (prosocial silence). Items are rated on a five‐point Likert scale ranging from 1 (*not at all*) to 5 (*very often*), where higher scores indicate higher levels of organisational silence. Kang and Go (2014) reported the following Cronbach's alpha values for the subsclaes: acquiescent silence, 0.87; defensive silence, 0.92; and prosocial silence, 0.85. In the current study, the Cronbach's alpha coefficients were 0.80 for acquiescent silence, 0.77 for defensive silence and 0.74 for prosocial silence.

### 4.3 Data Analysis

We initiated the data analysis by calculating descriptive statistics for the participants' demographic characteristics. Subsequently, Pearson's bivariate correlations were employed to assess relationships between key study variables. Using SPSS version 29.0, we also examined variance inflation factors, skewness and kurtosis to check for potential violations of normality or multicollinearity assumptions. Next, employing Mplus version 7.0, we evaluated the measurement model and hypothesised model using confirmatory factor analysis (CFA) and SEM. The robust maximum likelihood method was selected for model estimation. We assessed the model's adequacy using four goodness‐of‐fit indices: standardised root mean square residual (SRMR) < 0.08, comparative fit index (CFI) > 0.90, Tucker–Lewis index (TLI) > 0.90, and root mean square error of approximation (RMSEA) < 0.08 (Browne and Cudeck 1992). The SEM analyses were adjusted for years of nursing experience and unit tenure due to their significant correlations with key variables. Specifically, years of nursing experience were significantly correlated with acquiescent silence (*r* = −0.216, *p* = 0.001) and organisational citizenship behaviour (*r* = 0.278, *p* < 0.001), and unit tenure was significantly correlated with acquiescent silence (*r* = −0.158, *p* = 0.019). Furthermore, we investigated how authentic leadership indirectly influenced organisational citizenship behaviour through the three types of organisational silence. In doing so, we employed bootstrapping with 10,000 samples and 95% bias‐corrected confidence intervals (CI) were employed, as suggested by Preacher and Hayes (2008).

### 4.4 Ethical Considerations

This study was conducted in accordance with the Declaration of Helsinki and was approved by the Institutional Review Boards of Yonsei University Health System (#4‐2023‐0171; May 2, 2023) and Catholic University Saint Vincent's Hospital (#VC23QIDI0131; June 8, 2023). Data were collected anonymously, and participants voluntarily signed an informed consent form that explained the study purpose and methods, the assurance of anonymity, voluntary participation, the right to withdraw at any time without consequence, and the use of the study and participant data solely for research purposes.

## 5 Findings

### 5.1 Descriptive Statistics

As shown in Table 1, most participants were women (*n* = 200, 92.6%) and had a baccalaureate or higher degree in nursing (*n* = 205, 94.9%). Participants had a mean age of 31.7 years (standard deviation [SD] = 5.9) and mean nursing experience of 8.6 years (SD = 6.0), with an average of 5.0 years (SD = 3.9) working in their current unit. As for participants' hospital type, about 63.0% (*n* = 136) worked in a tertiary hospital and 37% (*n* = 80) in a general hospital.

**TABLE 1:** Participants' demographic characteristics (*N* = 216).

Characteristic	Category	*n* (%)	M (SD)
Gender	Women	200 (92.6)	
	Men	16 (7.4)	
Educational level	High school diploma	11 (5.1)	
	BSN or higher	205 (94.9)	
Age (years)			31.7 (5.9)
Nursing experience (years)			8.6 (6.0)
Unit tenure (years)			5.0 (3.9)
Hospital type	Tertiary	136 (63.0)	
	General	80 (37.0)	

Abbreviations: BSN, Bachelor of Science in Nursing; M, mean; SD, standard deviation.

### 5.2 Preliminary Analyses

Skewness and kurtosis values varied between −0.331 and 0.585 and did not exceed an absolute value of 2.0 (Curran et al. 1996), indicating no significant deviation from normality. Variance inflation factors between the predictors and mediators ranged from 1.148 to 1.887, indicating no problem with multicollinearity (Hair et al. 1998).

Table 2 presents Pearson's bivariate correlation coefficients and descriptive statistics for the key study variables. Authentic leadership was negatively correlated with acquiescent silence (*r* = −0.350, *p* < 0.001) and defensive silence (*r* = −0.240, *p* < 0.001) and positively correlated with prosocial silence (*r* = 0.201, *p* = 0.003) and organisational citizenship behaviour (*r* = 0.298, *p* < 0.001). Acquiescent silence was positively correlated with defensive silence (*r* = 0.612, *p* < 0.001) and negatively correlated with prosocial silence (*r* = −0.327, *p* < 0.001). The correlation between defensive silence and prosocial silence was nonsignificant (*r* = −0.110, *p* = 0.107). Organisational citizenship behaviour was negatively correlated with acquiescent silence (*r* = −0.526, *p* < 0.001) and defensive silence (*r* = −0.396, *p* < 0.001) and positively correlated with prosocial silence (*r* = 0.320, *p* < 0.001).

**TABLE 2:** Correlations, means and standard deviations for key study variables (*N* = 216).

Variable	1	2	3	4	5
1. Authentic leadership	—				
2. Acquiescent silence	−0.350***	—			
3. Defensive silence	−0.240***	0.612***	—		
4. Prosocial silence	0.201**	−0.327***	−0.110	—	
5. Organisational citizenship behaviour	0.298***	−0.526***	−0.396***	0.320***	—
M	3.359	2.124	2.182	3.767	3.909
SD	0.715	0.608	0.760	0.640	0.374

Abbreviations: M, mean; SD, standard deviation.

** *p* < 0.01.

*** *p* < 0.001.

### 5.3 Hypothesis Testing

We conducted CFA to evaluate the performance of the five‐factor measurement model and to determine how well the observed items corresponded with their latent variables. To enhance the precision of parameter estimates and minimise sampling errors and bias, parcels were created for the authentic leadership and organisational citizenship behaviour scales, which contained more than four items (Little et al. 2002). Following the internal consistency approach (Little et al. 2002), the 16 authentic leadership items and 20 organisational citizenship behaviour items were each grouped into four and five parcels, respectively, reflecting the facet structure of the original scales.

Based on the fit index values, the model demonstrated a good fit to the data: *χ* ^2^ (199) = 368.807, CFI = 0.923, TLI = 0.911, RMSEA = 0.063 and SRMR = 0.052. Standardised loadings varied from 0.416 to 0.930, all of which were statistically significant (*p* < 0.001). In an alternative model, the three subscales of organisational silence (acquiescent silence, defensive silence and prosocial silence) were consolidated into a single variable, which resulted in poorer fit index values: *χ* ^2^(206) = 740.664, CFI = 0.759, TLI = 0.730, RMSEA = 0.110 and SRMR = 0.092. The Akaike information criterion values further supported the original model, with the alternative model scoring 9152.601, which was higher than the original model's 8794.743 (Burnham and Anderson 2004); these values suggested that maintaining the three subscales of organisational silence as separate variables offered greater validity.

Additionally, we compared partial and full mediation models to determine which better fit the data. The partial mediation model showed a moderately good fit to the data: *χ* ^2^ (242) = 470.166, CFI = 0.899, TLI = 0.885, RMSEA = 0.066 and SRMR = 0.082. The full mediation model, which excluded the direct path from authentic leadership to organisational citizenship behaviour, demonstrated a reasonably good fit: *χ* ^2^(243) = 470.376, CFI = 0.899, TLI = 0.886, RMSEA = 0.066 and SRMR = 0.082. A chi‐squared difference test revealed that adding a direct path between authentic leadership and organisational citizenship behaviour did not significantly improve the model fit (∆*χ* ^2^ = 0.210, ∆df = 1, *p* > 0.05), indicating that the full mediation model was more appropriate.

**FIGURE 1:**
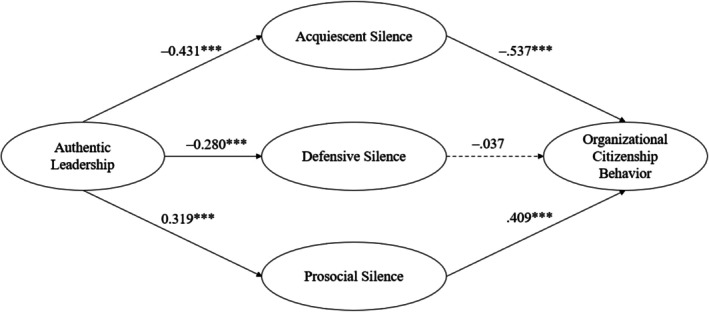
Structural equation model of the hypothesised mediation model with standardised coefficient estimates. Nonsignificant paths are specified by a dotted line. ***p < 0.001.

As illustrated in Figure 1, authentic leadership significantly reduced both acquiescent silence (*β* = −0.431, *p* < 0.001) and defensive silence (*β* = −0.280, *p* < 0.001) and significantly increased prosocial silence (*β* = 0.319, *p* < 0.001). The effects of the three mediators on organisational citizenship behaviour varied. Acquiescent silence and prosocial silence influenced organisational citizenship behaviour in opposite directions (*β* = −0.537, *p* < 0.001 for acquiescent silence; *β* = 0.409, *p* < 0.001 for prosocial silence), whereas no significant relationship was found between defensive silence and organisational citizenship behaviour (*β* = −0.037, *p* = 0.749).

According to Preacher and Hayes (2008), the distribution of indirect effects frequently exhibits skewness. To address this issue, 95% bias‐corrected confidence intervals (95% CI) were bootstrapped utilising 10,000 samples derived from the original data. We found that two of the three hypothesised mediation effects were significant (Table 3). Specifically, the indirect effects of authentic leadership on organisational citizenship behaviour through acquiescent silence (*β* = 0.232, 95% CI [0.071, 0.393]) and prosocial silence (*β* = 0.131, 95% CI [0.051, 0.210]) were both statistically significant, as the 95% CIs did not include zero. Conversely, defensive silence did not significantly mediate the relationship (*β* = 0.010, 95%, CI [−0.085, 0.106]).

**TABLE 3:** Standardised indirect effects for the hypothesised mediation model (*N* = 216).

Path	*β*	SE (*b*)	*p*	95% CI	
				Lower	Upper
Authentic leadership ➔ acquiescent silence ➔ organisational citizenship behaviour	0.232	0.082	0.011	0.071	0.393
Authentic leadership ➔ defensive silence ➔ organisational citizenship behaviour	0.010	0.049	0.835	−0.085	0.106
Authentic leadership ➔ prosocial silence ➔ organisational citizenship behaviour	0.131	0.041	0.002	0.051	0.210

Abbreviations: CI, confidence interval; SE, standard error.

## 6 Discussion

This study examined the relationship between nurse managers' authentic leadership and staff nurses' organisational citizenship behaviour and investigated whether this relationship was mediated by three types of organisational silence: acquiescent, defensive and prosocial. The findings provide compelling evidence for the complex interplay between authentic leadership and nurses' discretionary behaviours in the healthcare context.

Our results supported Hypothesis 1, which contended that authentic leadership would be positively associated with organisational citizenship behaviour. This finding aligns with results of previous research (Farid et al. 2020) indicating that authentic leaders foster environments where employees feel valued and motivated to exceed their contractual obligations. The positive relationship between authentic leadership and organisational citizenship behaviour underscores the critical role of leadership in promoting employee behaviours that enhance organisational effectiveness. This finding is particularly relevant in dynamic environments such as healthcare, where both complex tasks and the need for collaboration necessitate leadership that fosters trust and motivation.

Moreover, we found that authentic leadership is negatively associated with acquiescent silence and defensive silence, supporting Hypothesis 2. May et al. (2003) suggest that authentic leaders who value communication and diverse perspectives inspire employees to constructively engage within the organisation. Such engagement may lead employees to perceive themselves as agents of constructive change, viewing the act of speaking up and breaking silence as positive and beneficial behaviour (Xu et al. 2023). In addition, previous research (Akuzum 2014) indicated that perceptions of fairness in organisational decision‐making are associated with lower levels of acquiescent and defensive silence. These findings highlight the potential of authentic leadership to reduce these forms of organisational silence and foster a culture where employees feel empowered to share their perspectives.

As proposed in Hypothesis 2, authentic leadership was positively associated with prosocial silence. Leaders who emphasise ethical behaviour and respect create an environment where employees are encouraged to protect the organisation's interests. In this context, prosocial silence may reflect employees' deliberate choice to withhold sensitive information or avoid raising issues with a disruptive, compromising impact on organisational harmony and confidentiality (Kızrak and Yeloğlu 2024). Employees who perceive their leaders as trustworthy and morally upright are more likely to reciprocate by engaging in behaviours that prioritise the well‐being of the organisation (Farid et al. 2020). However, more research is needed to examine how specific aspects of authentic leadership, such as ethical transparency or interpersonal respect, shape prosocial silence among nurses.

Additionally, our results indicated that different types of silence were differentially associated with organisational citizenship behaviour. Specifically, acquiescent silence negatively affected organisational citizenship behaviour, which supports Hypothesis 3 and is consistent with previous studies (Acaray and Akturan 2015; Go and Cho 2020). This disengaged form of silence, characterised by withholding opinions due to resignation (Van Dyne et al. 2003), likely leads to decreased engagement in organisational citizenship behaviour (Hao et al. 2022). Our results support previous research indicating that silent employees exhibited low organisational citizenship behaviour (Mao et al. 2019). Contrary to Hypothesis 3, defensive silence, which is motivated by self‐protection based on fear of the consequences of speaking up, was not significantly related to organisational citizenship behaviour. This finding contradicts earlier studies reporting that defensive silence negatively affects organisational citizenship behaviour (Go and Cho 2020). However, it supports the idea that while employees may withhold opinions to avoid conflict, this does not necessarily mean that they disengage from organisational citizenship behaviour (Xu et al. 2023). Therefore, self‐defensive silence may be seen as a strategy to avoid unfavourable situations and outcomes (Xu et al. 2023) without detaching from organisational citizenship behaviour.

Prosocial silence was positively associated with organisational citizenship behaviour, supporting Hypothesis 3 and consistent with previous findings reported by Acaray and Akturan's (2015). Given its underlying intention to benefit the organisation or protect sensitive information (Van Dyne et al. 2003), prosocial silence generally appears to foster engagement in organisational citizenship behaviour. These findings may be especially salient in the South Korean healthcare context, where strong hierarchical structures and collectivist norms (Lee and Lee 2024) may render prosocial silence a culturally compatible form of constructive restraint that facilitates organisational citizenship behaviour. However, excessive prosocial silence may have adverse effects, as employees may avoid challenging supervisors or questioning the status quo, behaviours that could hinder organisational learning (Shahjehan and Yasir 2017). This underscores the need for further research to explore the nuanced relationship between prosocial silence and organisational citizenship behaviour in nursing contexts.

Our findings revealed that the relationship between authentic leadership and organisational citizenship behaviour was mediated by both acquiescent and prosocial silence, partially supporting Hypothesis 4. Authentic leaders appear to encourage nurses to shift away from acquiescent silence to active engagement, which, in turn, increases their organisational citizenship behaviour. This finding supports the notion that, although nurses may not initially feel strong ties to their organisation, honest communication and genuine leadership from managers can foster stronger connections among employees and motivate them to contribute to organisational goals (May et al. 2003). Furthermore, authenticity on the part of managers seems to promote prosocial silence among nurses, facilitating the smooth operation of the organisation by encouraging employees to act in the organisation's best interests. However, contrary to our hypothesis, defensive silence did not mediate the relationship between authentic leadership and organisational citizenship behaviour. This finding may indicate that defensive silence is primarily driven by fear of receiving negative feedback (Van Dyne et al. 2003), which authentic leadership alone may not fully mitigate. Although authentic leadership may help reduce defensive silence by fostering trust and psychological safety, this reduction does not appear to directly contribute to organisational citizenship behaviour. Future research should further explore the complex mechanisms underlying defensive silence and its potential links to leadership and organisational outcomes.

### 6.1 Limitations and Recommendations for Future Research

This study has some limitations. First, data were collected from nurses working in five hospitals in South Korea, which may limit the generalisability of the findings to other cultural or organisational contexts. Future research should replicate this study in diverse settings to validate the results and extend their applicability. Second, all variables were measured using self‐report questionnaires administered to the same respondents at a single point in time, which raises concerns of common method bias. While we assured participants of anonymity and confidentiality to reduce potential response biases, such as social desirability, these procedures may not fully prevent inflation of the observed associations due to shared method variance. Future research should consider employing alternative measurement approaches or data sources to reduce this bias and enhance the validity of the findings. Third, participants' responses reflected their subjective evaluations of leadership, which may not accurately capture actual leader behaviours. As Fischer et al. (2024) point out, commonly used leadership style measures often conflate observed behaviours with evaluative judgements, leading to potential causal illusions. Future research should aim to distinguish between perceptions and behaviours, possibly through multi‐source or behavioural data. Finally, the study's cross‐sectional design precluded drawing of causal inferences. Future research could use longitudinal designs to capture the temporal dynamics of leadership behaviours and their impact on employees' silence and citizenship behaviour over time.

### 6.2 Implications for Policy and Practice

Our study provides practical insights for nursing management, particularly by confirming that nurse managers' authentic leadership significantly impacts staff nurses' propensity for organisational silence. Nurses who perceive their managers as authentic are more likely to view them positively (Xu et al. 2023) and feel motivated to contribute to organisational success (Zhang et al. 2018). To leverage these findings, hospital management should implement training and development programmes to help nurse managers cultivate authentic leadership behaviours (Farid et al. 2020). Furthermore, nurse managers should work to foster an organisational culture that values diverse perspectives and promotes psychological safety, ensuring that nurses feel comfortable speaking up without fear of judgement or adverse consequences (Yamak and Eyupoglu 2021). Such an environment can encourage nurses to share their opinions and ideas, ultimately enhancing their organisational citizenship behaviour and improving the overall effectiveness of the healthcare system (Acaray and Akturan 2015).

## 7 Conclusions

This study demonstrated that authentic leadership among nurse leaders plays a crucial role in promoting staff nurses' organisational citizenship behaviour by reducing acquiescent and defensive silence, which can otherwise undermine nurses' engagement and commitment to their organisation. Additionally, trust in leaders' moral and ethical integrity can foster prosocial silence, encouraging nurses to withhold feedback in ways that preserve harmony and contribute to collaborative and constructive behaviours among staff. However, while authentic leadership supports organisational harmony, leaders must also promote open communication and encourage nurses to voice their ideas and concerns. By balancing support for prosocial silence with openness and advocacy, nurse leaders can cultivate an organisational culture that drives both professionalism and excellence in healthcare delivery.

## Author Contributions

H.J.L., J.K.S., S.E.L.: made substantial contributions to conception and design, or acquisition of data, or analysis and interpretation of data; given final approval of the version to be published. Each author have participated sufficiently in the work to take public responsibility for appropriate portions of the content; agreed to be accountable for all aspects of the work in ensuring that questions related to the accuracy or integrity of any part of the work are appropriately investigated and resolved.

## Conflicts of Interest

The authors declare no conflicts of interest.

## Supporting information


Data S1.

## Data Availability

The data that support the findings of this study are available on request from the corresponding author. The data are not publicly available due to privacy or ethical restrictions.
